# Laser excision of a large granular cell tumor of the vocal cord with subglottic extension: A case report

**DOI:** 10.1515/med-2025-1250

**Published:** 2025-08-07

**Authors:** Manal Bukhari

**Affiliations:** Department of Otolaryngology-Head and Neck Surgery, College of Medicine, King Saud University, Riyadh, Saudi Arabia

**Keywords:** Abrikossoff’s tumor, dysphonia, granular cell tumor, laryngeal neoplasm

## Abstract

Granular cell tumors (GCTs) are rare, benign tumors typically originating from Schwann cells, with the head and neck being the most common sites. Laryngeal GCTs, particularly those affecting the vocal cords, are exceedingly rare in adults. This report presents a 28-year-old female with a GCT of the left vocal cord extending into the subglottic region. The patient presented with progressive dysphonia, and laryngoscopy revealed a 2 cm × 1 cm mass on the left vocal cord. Microlaryngeal examination confirmed subglottic extension, and the tumor was excised using carbon dioxide (CO_2_) laser. Histopathological analysis confirmed GCT with S100 positivity. Two months later, the patient developed a late complication – granuloma formation at the excision site – necessitating revision surgery. The patient was symptom-free at the 12-month follow-up. GCTs in the vocal cords with subglottic extension are rare and challenging to diagnose and treat. They are generally benign lesions and rarely undergo malignant transformation. Diagnosis is confirmed through histology, and treatment involves wide local excision, with re-excision needed for recurrence. GCTs are chemo- and radio-insensitive, making surgery the primary treatment. This case underscores the importance of accurate diagnosis and tailored treatment, highlighting the need for further research on this rare condition.

## 1 Introduction

Granular cell tumors (GCTs) are extremely rare, benign neoplasms of soft tissue that likely originate from Schwann cells [1]. First described by Abrikossoff in 1926, these tumors are also known as Abrikossoff’s tumors [2]. Approximately 50% of all GCTs occur in the head and neck region, with the tongue being the most frequently implicated organ, accounting for nearly 30% of all cases. Less than 10% of cases involve the larynx [3]. Laryngeal GCTs, particularly those affecting the vocal cords, are exceptionally rare, with the majority of cases occurring in the adult age group [3]. The occurrence of GCTs in the vocal cords of adults is even rarer, underscoring the need for further research to gather more data, which will be crucial for advancing our understanding and management of this uncommon entity. Here, we present the case of a GCT of the vocal cords in a 28-year-old woman.

## 2 Case report

A 28-year-old female presented to the otolaryngology clinic with a complaint of progressive dysphonia over the past year. She denied any history of dyspnea, dysphagia, or choking and had no previous history of allergies or surgery. She is a non-smoker and does not consume alcohol.

**Figure 1:**
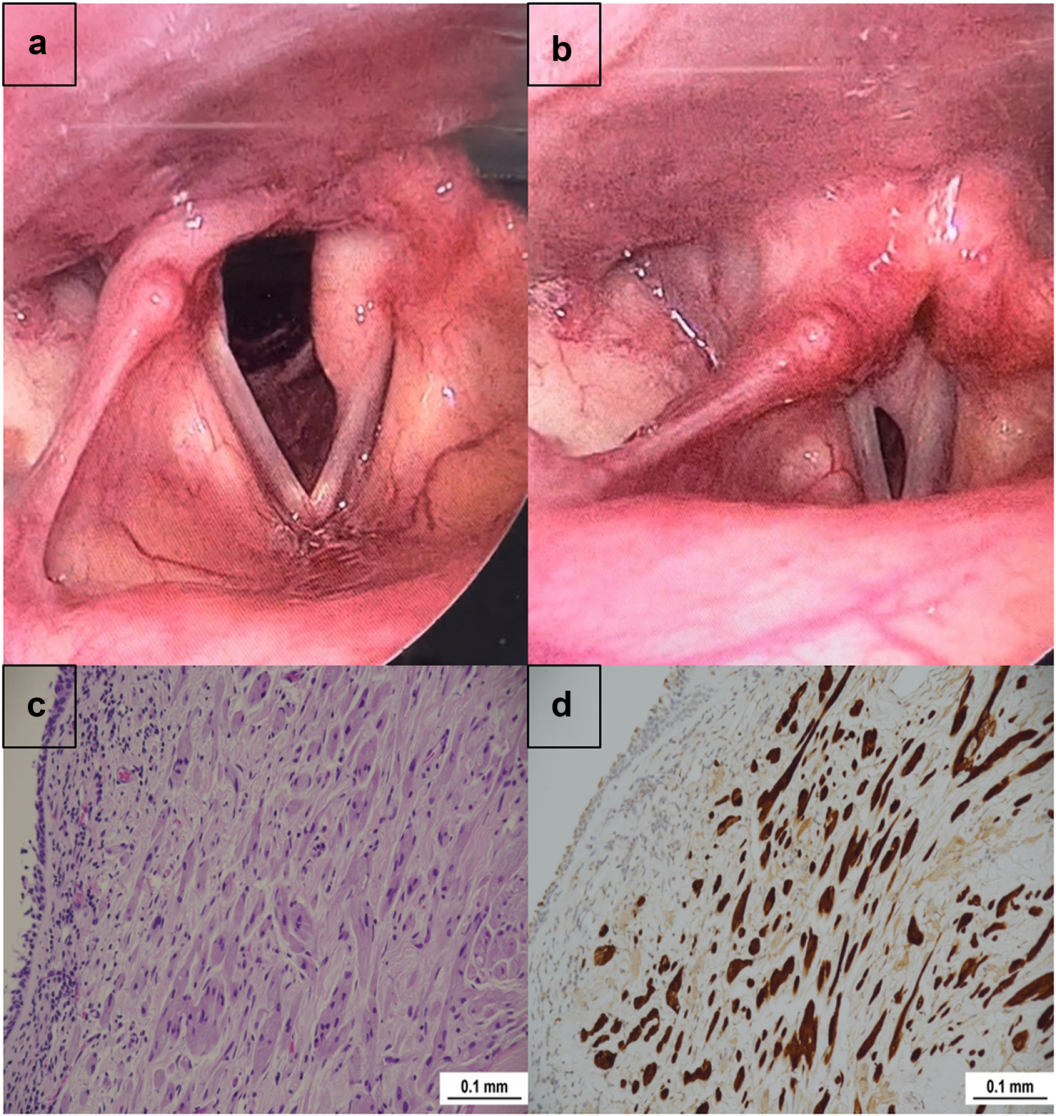
GCT of the vocal cord. (a) Direct laryngoscopy revealing a 2 × 1 cm mass with a smooth surface and whitish coloration in the posterior two-thirds of the left vocal cord. (b) Direct laryngoscopy revealing a 2 mm anterior glottic gap during phonation, and there was mass effect. (c) Light microscopy image of the resected vocal cord tumor showing polygonal cells with abundant eosinophilic cytoplasm (hematoxylin and eosin stain; ×20). (d) Immunohistochemical staining of the resected vocal cord tumor showing nuclear and cytoplasmic positivity for S100 (×20).

A direct laryngoscopy revealed a 2 cm × 1 cm mass with a smooth appearance and whitish coloration in the posterior two-thirds of the left vocal cord (Figure 1a). Both vocal cords were mobile with a 2 mm anterior glottic gap during phonation, and there was mass effect (Figure 1b). A microlaryngeal examination under general anesthesia revealed that the mass extended into the subglottic area. The mass was firm on palpation and sheltered by epithelium. A complete resection of the mass was achieved using a carbon dioxide (CO_2_) laser.

The patient was discharged the day post-surgery with no intraoperative or early postoperative complications. She was instructed to rest her voice for 5 days. During the first follow-up (1 week later), the patient’s voice had significantly improved. A follow-up laryngoscopy showed whitish slough tissue at the posterior third of the glottis. Histopathological results revealed a mass covered by non-keratinizing stratified squamous epithelium with large polygonal cells containing granular cytoplasm (Figure 1c). S-100 staining was positive (Figure 1d).

**Figure 2:**
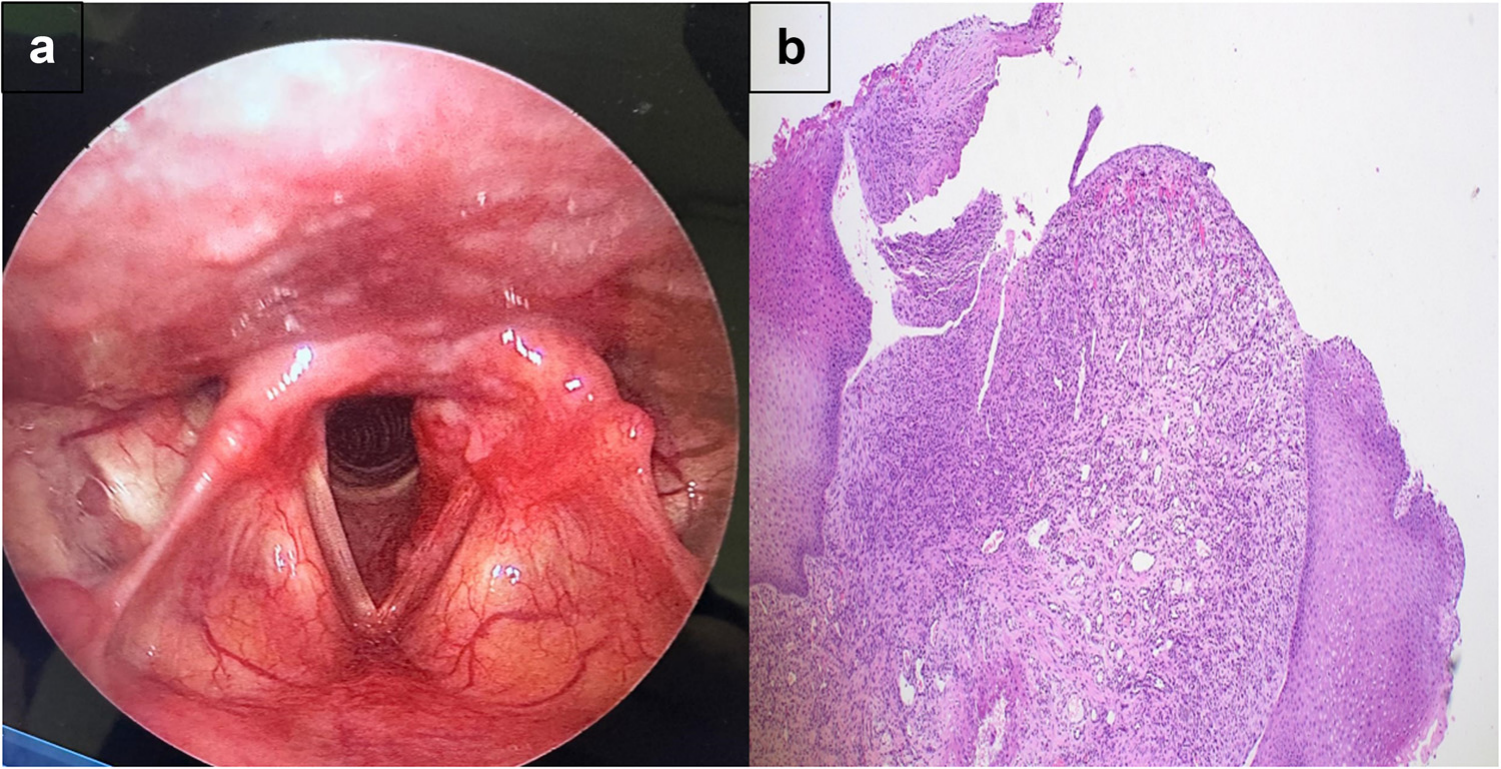
Recurrent mass of the vocal cord. (a) Direct laryngoscopy revealing a 2 × 0.5 cm mass with an irregular, granular surface and red coloration, extending from the posterior infraglottic area to the posterior two-thirds of the vocal cord. (b) Light microscopy image of the recurrent vocal cord mass showing surface ulceration with underlying lobular arrangement of capillary vessels (hematoxylin and eosin stain; ×40).

Two months later, the patient developed dysphonia again. A direct laryngoscopy revealed a 2 cm × 0.5 cm mass with an irregular, granular surface and red coloration, extending from the posterior infraglottic area to the posterior two-thirds of the vocal cord (Figure 2a). Revision surgery was performed using a CO_2_ laser to remove the mass, with intraoperative local steroid injection at the surgery site. The patient was discharged the next day and instructed to rest her voice for 1 week, take oral corticosteroids for 1 week, and use a proton pump inhibitor for 1 month. Histology showed intense inflammatory cell infiltrate with blood vessel proliferation, suggesting granuloma (Figure 2b), but was negative for GCT.

**Figure 3:**
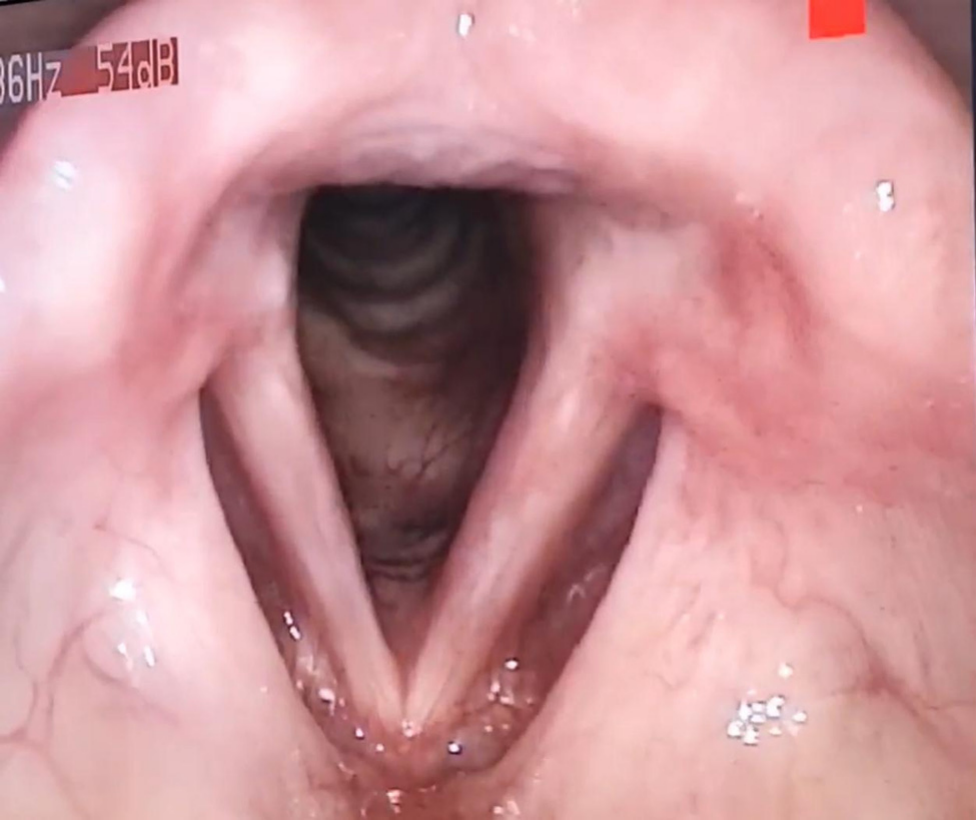
Direct laryngoscopy demonstrating remarkable healing of the left vocal cord, with no evidence of mass recurrence 1 year after surgery.


Two months later, the patient’s voice was clear, and follow-up laryngoscopy showed complete closure during phonation with good healing. Twelve months after the last surgery, the patient was symptom-free and had no recurrence (Figure 3).

**Informed consent**: Written informed consent was obtained from the patient for publication of this case report and accompanying images.
**Ethical approval**: Ethical approval for this case report was waived from the Institutional Review Board at King Saud University, Riyadh, Saudi Arabia.

## 3 Discussion

GCTs are known by several other names, including Abrikossoff’s tumor, granular cell nerve sheath tumor, granular cell schwannoma, and granular cell myoblastoma [2]. Epidemiologically, GCTs are believed to be more prevalent in adults [4], females [3], and individuals of African descent [5].

While GCTs can affect any part of the body, laryngeal involvement is not common [3]. Patients with laryngeal GCTs may be asymptomatic. However, those who do experience symptoms may report hoarseness, cough, difficulty swallowing, painful swallowing, ear pain, a high-pitched breathing sound, or coughing up blood [3]. In our study, the patient presented with voice changes but had no history of shortness of breath or difficulty swallowing.

GCTs typically present as solitary, well-circumscribed, firm, pink, or greyish-yellow masses [4]. Multifocality may suggest the presence of syndromes like Noonan syndrome or neurofibromatosis type I [4]. GCTs of the vocal cords most commonly affect the anterior one-third. In contrast, our patient had involvement of the posterior two-thirds with subglottic extension, a presentation that is rarely reported. GCT of the vocal cord with subglottic extension is clinically significant due to its impact on diagnosis, symptoms, treatment, and prognosis. Subglottic involvement can complicate diagnosis and treatment, potentially affecting the airway and leading to symptoms such as difficulty breathing, stridor, or dysphagia. It may also require more invasive treatments. The extension into the subglottis may necessitate a more extensive surgical resection, with risks of airway compromise or recurrence if not fully excised.

The histogenesis of GCTs has been widely debated. Our understanding has evolved since Abrikossoff’s 1926 description, in which he proposed a myogenous origin [2]. Later evidence in 1962, however, reinforced a neural source, linking these neoplasms to Schwann cells [1]. Features such as S100 protein positivity, neuron-specific enolase, the similarity between myelin granules and those in GCT cells, and the concentric organization of granular cells positioned around nerve endings corroborate the neural origin hypothesis. Additionally, the presence of lipoproteins and sphingomyelin further suggests a Schwann cell origin [1].

Although the majority of GCTs are benign, less than 2% of cases could undergo malignant transformation and exhibit aggressive biological behavior [6]. The differential diagnoses of GCTs include polyps, granulomas, and cysts, which are often difficult to distinguish through direct visualization alone. Therefore, a definitive diagnosis is typically made through histological examination of biopsy samples obtained during direct laryngoscopy. Microscopically, GCTs display a distinctive appearance, with hematoxylin-eosin staining revealing plentiful eosinophilic, granular cytoplasm. Immunohistochemical analysis for S100 and neuron-specific enolase positivity may be necessary to confirm the diagnosis [7].

Treatment of GCTs of the vocal cords involves wide local excision with the aim of achieving negative margins. Carbon dioxide (CO_2_) laser excision has proven to be an effective method for removing the tumor with negative margins [8]. Recurrence is largely rare. GCTs are resistant to chemotherapy and radiotherapy [9], so re-excision is preferred in case of relapse. Small-sized GCTs can be excised via direct laryngoscopy, while larger ones require laryngofissure or more radical approaches like laryngectomy.

## 4 Conclusion

GCTs of the vocal cords are extremely rare benign neoplasms, with the majority occurring in the adult population. Laryngeal GCTs, especially those involving the vocal cords and subglottic extension, present diagnostic and therapeutic challenges due to their potential airway impact and the need for extensive surgical resection. While GCTs are typically benign, malignant transformation is possible in a small percentage of cases. Diagnosis is confirmed through histological examination, with S100 and neuron-specific enolase staining aiding in identification. Treatment involves wide local excision, with re-excision recommended for recurrences, as these tumors are resistant to chemotherapy and radiotherapy. Further research is needed to better understand and manage this rare entity.
